# The Correlation Between Serum Fibroblast Growth Factor 21 and the Severity and Occurrence of Coronary Artery Disease

**DOI:** 10.7759/cureus.51924

**Published:** 2024-01-09

**Authors:** Seema R Sinha, Prem Prakash, J. R Keshari, Ravi V Prasad

**Affiliations:** 1 Biochemistry, Indira Gandhi Institute of Medical Sciences, Patna, IND; 2 General Surgery, Indira Gandhi Institute of Medical Sciences, Patna, IND; 3 Biochemistery, Indira Gandhi Institute of Medical Sciences, Patna, IND; 4 Cardiology, Indira Gandhi Institute of Medical Sciences, Patna, IND

**Keywords:** coranary artery disease, syntax score, gensini score, stable angina pectoris, fgf21

## Abstract

Background: The burden of cardiovascular diseases (CVDs) is increasing worldwide with CVD being one of the leading causes of death, including atherosclerosis, myocardial infarction, cardiomyopathy, and heart failure (HF). Fibroblast growth factor 21 (FGF21) is an endocrine hormone that regulates carbohydrate and lipid metabolism. It exerts direct effects on the cardiovascular system and can serve as an early indicator of CVDs. FGF21's therapeutic properties include reducing obesity, dyslipidaemia, and hyperglycemia, which can help treat metabolic disorders, autophagy, and apoptosis. Atherosclerosis is developed due to chronic inflammatory conditions, and the immune system's reaction to oxidized lipoproteins is mainly responsible for the development of atherosclerosis. FGF21's precise role in the pathogenesis of coronary artery disease (CAD) remains elusive.

Aim: This study aimed to assess the role of FGF21 in predicting the severity and magnitude of CAD in individuals diagnosed with stable angina pectoris (SAP).

Materials and methods: A prospective cross-sectional study was conducted on 110 consecutive patients with SAP reported to the cardiology department of the Indira Gandhi Institute of Medical Sciences (IGIMS), Patna, India. They were divided into two groups based on coronary angiography findings. Control groups included patients not showing any atherosclerotic lesions and case groups with atherosclerotic lesions. The SYNTAX score is a grading system that measures the location and complexity of coronary arteries using anatomical principles. The Gensini score assessment technique was employed to determine the severity of CAD. We compared serum FGF21 levels,left ventricular ejection fraction (LVEF), and inflammatory biomarker C-reactive protein (CRP) levels between the two groups. Moreover, we examined the correlation between the serum FGF21 level and the SYNTAX and Gensini scores. The statistical analysis was done using Version 23.0 of SPSS Statistics. P-values below 0.05 were considered statistically significant.

Results: The study found that the case group had a higher average age and a higher proportion of male patients. The case group had considerably higher levels of FGF21 (166.59 ± 94.49791 pg/mL) compared to the control group (54.13 ± 48.467 pg/mL) (p=0.034). The LVEF exhibited a significant difference between the case and control groups, with mean values of 50.3056 ± 7.8242% and 56.078 ± 5.3987%, respectively (p=0.031). CRP levels were comparable in both groups. The case group had mean values of SYNTAX and Gensini scores of 23.19±7.43 and 50.03±27.30, respectively. We found that there was no statistically significant association between the risk assessments for CAD severity and the levels of serum FGF21 (correlation coefficient r=0.14070, p>0.05, and r=0.206415, p>0.05, respectively)

Conclusions: FGF21 is gaining recognition as a prospective addition to the FGF family, potentially playing a significant role in cardiovascular disease, particularly atherosclerosis. A statistically significant difference was seen in the serum FGF21 levels between the case and control groups, indicating that it can help in the diagnosis of CAD. However, there was no apparent correlation found between the serum FGF21 levels and the SYNTAX and Gensini scores. The role of FGF21 in the development of atherosclerosis and whether FGF21 could serve as a reliable marker need to be studied further.

## Introduction

The prevalence of cardiovascular diseases (CVDs) is escalating globally, posing a significant risk to human life and well-being. Fibroblast growth factor 21 (FGF21) is a member of the FGF family that functions as an endocrine hormone that regulates carbohydrate and lipid metabolism. FGF21 exerts direct effects on the cardiovascular system (CVS) and may be useful as an early biomarker for CVDs [1]. CVDs are among the primary contributors to mortality, encompassing conditions such as atherosclerosis, myocardial infarction, cardiomyopathy, and heart failure (HF) [1]. The primary regulators of FGF21 in the liver and adipose tissue are peroxisome proliferator-activated receptor (PPAR)α and PPARγ, respectively. In target tissues, it is capable of stimulating homologous FGF receptors (FGFRs) when the coreceptor β-klotho is present. By modulating oxidative stress, lipid metabolism FGF21 has therapeutic properties for a wide range of human ailments, including alopecia, diabetes mellitus, and renal disease. Its therapeutic properties reduce obesity, dyslipidemia, and hyperglycemia, thereby dealing with metabolic disorders, autophagy, and apoptosis. FGF21 potentially serves as a novel target for the prediction and treatment of cardiovascular diseases. Atherosclerosis is developed and seen due to chronic inflammatory conditions, and the immune system's reaction to oxidized lipoproteins is mainly responsible for triggering the process of atherosclerosis development [2,3]. There are various inflammatory biomarkers, particularly cytokines, that have been studied for their potential to prevent and detect CVD at an early stage [4-6]. A recent study from Saudi Arabia concluded that FGF-21 can also be used as a marker to predict non-alcoholic fatty liver disease in people with diabetes mellitus type 2 due to its high sensitivity and specificity compared to the other markers [7]. While several researchers have demonstrated a relationship between FGF21 and CVD, its precise role in the pathogenesis of coronary artery disease (CAD) remains unknown [8]. Our objective was to evaluate the involvement of FGF21 in the development of CAD and the severity of CAD in patients with stable angina pectoris (SAP).

## Materials and methods

Patient population

A cross-sectional study was carried out among adult patients with SAP who underwent coronary angiography at the cardiology department, and the blood investigations were done in the department of biochemistry at the Indira Gandhi Institute of Medical Sciences (IGIMS), a tertiary care hospital located in Patna, Bihar, India. The study was carried out between April 2023 and November 2023. Ethical approval was obtained from the Ethics Committee of IGIMS, vide 913/IEC/IGIMS/2023. All the consecutive patients with symptoms of SAP, i.e., the occurrence of characteristic chest discomfort or similar symptoms during physical activity, along with a positive treadmill test or identification of ischemia under stress, were enrolled in this study after taking proper informed consent. Exclusion criteria for this study were patients having a history of previous acute coronary syndrome (ACS), documented CAD, autoimmune diseases, malignancies, severe valvular diseases of the heart, a left ventricular ejection fraction (LVEF) of <50%, chronic or inflammatory systemic diseases, chronic kidney disease, hematological disorders, thyroid dysfunctions, pregnancy, and suspicious pericarditis or myocarditis. A detailed clinical history was obtained in all cases: the age of the subject, sex, present and past history, history of cigarette smoking, history of hypertension, diabetes mellitus, and tuberculosis were recorded before echocardiography and myocardial perfusion scintigraphy. All patients underwent a thorough two-dimensional transthoracic echocardiography evaluation. The bi-planar Simpson method was used to calculate the LVEF. Coronary angiography was done in all suspected cases of SAP. Individuals who did not have any atherosclerotic lesions on coronary angiography were categorized as the control group, and individuals who had atherosclerotic lesions were grouped as cases.

Blood samples

After eight to 12 hours of overnight fasting, venous blood samples from the patients were collected. Blood was allowed to clot by placing the tube in a rack at room temperature for at least 30 minutes. Then it was centrifuged at 2000 rpm for 10 minutes. Within two hours of centrifugation, the serum was transferred by pipette into another tube and tightly stopped. The clear serum was analyzed within 8 hours if kept at room temperature or stored at 2-8°C if not analyzed the same day. For more than 48 hours of storage at -20°C, a refrigerator was used. Frozen samples were thawed only once for analysis. Fasting blood glucose (FBG), serum creatinine, lipid profile, and inflammatory marker C-reactive protein (CRP) levels were analyzed on a Beckman Coulter AU5800 Clinical Chemistry auto-analyzer by the chemiluminescence method. Serum FGF21 was measured using the ELISA kit method. The absorbance was measured at 450 nm with an Alere AM 2100 microplate reader. The range of the assay was 5 pg/mL to 1,500 pg/mL. All the biochemical parameters were compared between the case and control groups.

Coronary angiography

Coronary angiography was done in the cardiac cath lab of the cardiology department at IGIMS. Patna digital angiographic devices were used to capture angiographic images of the coronaries. Significant CAD was characterized as a reduction of the diameter of the blood vessel by more than 50%, encompassing the three primary coronary arteries and the initial branches of the left anterior descending artery, or circumflex artery. The diagnostic angiograms were captured using a digital media viewer and analyzed by skilled cardiac specialists.

Atherosclerotic lesion severity scoring

Atherosclerotic lesion severity was assessed using the two scoring systems, namely SYNTAX scores and Gensini scores. The SYNTAX score is a scoring system that uses anatomical criteria to quantitatively assess coronary arteries in terms of the quantity, complexity, location, and functional nature of angiographically obstructive lesions. Each lesion with a stenosis diameter of ≥50% in arteries measuring 1.5 mm or larger is evaluated using coronary angiography. A weight factor is provided to each coronary segment based on the location and severity of the related lesions. The features of the lesions, such as complete blockage, division into three branches, division into two branches, presence of calcium deposits, twisted shape, length exceeding 20 mm, blood clot, and widespread or small-vessel disease, are combined using the online SYNTAX calculator (www.syntaxscore.com) to calculate the ultimate score [9, 10]. The score serves as an impartial angiographic instrument for evaluating the intricacy of CAD and determining its extent. The final online updated version (2.28) of SYNTAX Working Group 2020 was used.

The Gensini score assessment technique was used to measure the severity of CAD. The severity of stenosis and the location of the coronary artery lesion were assessed using the following scoring system: 1 point is awarded for narrowing of ≤25%, 2 points for narrowing of 26-50%, 4 points for narrowing of 51-75%, 8 points for narrowing of 76-90%, 16 points for narrowing of 91-99%, and 32 points for total occlusion. Subsequently, each lesion score is multiplied by a factor that considers the significance of the lesion's position in the coronary circulation. This factor is 5 for the left main coronary artery, 2.5 for the proximal segment of the left anterior descending coronary artery, 2.5 for the proximal segment of the circumflex artery, 1.5 for the mid-segment of the left anterior descending coronary artery, 1.0 for the right coronary artery, the distal segment of the left anterior descending coronary artery, the posterolateral artery, and the obtuse marginal artery, and 0.5 for other segments. Ultimately, the Gensini score was determined by adding up the scores of each individual coronary section [11].

Statistical analysis

Version 23.0 of SPSS Statistics was used for the statistical analysis. The Shapiro-Wilk test was utilized to evaluate the normal distribution of quantitative data, and it was established that variables and subgroups adhered to the normality assumption. The chi-square test was used for categorical variables, whereas the independent sample t-test was used for continuous variables. To assess the correlation between continuous data, the Pearson correlation was employed. An examination of logistic regression was used to determine the relationship between FGF21, the SYNTAX score, and the Gensini score. P-values less than 0.05 were deemed statistically significant.

## Results

The study enrolled a total of 110 subjects, of which 70 were in case groups (patients with SAP having atherosclerotic lesions on angiography) and 40 were in the control group (patients with SAP without any atherosclerotic lesions on angiography). The demographics, laboratory parameters, clinical characteristics, and other factors of the groups are depicted in Table 1.

**Table 1:** Baseline demographics – clinical characteristics, laboratory, and angiographic parameters. FSG, fasting serum glucose; WBC, white blood cells; TChol, total cholesterol; LDL-C, low-density lipoprotein cholesterol; HDL-C, high-density lipoprotein cholesterol; TG, triglycerides; CRP, C-reactive protein; FGF21, fibroblast growth factor 21; LVEF, left ventricular ejection fraction, SAP: stable angina pectoris. The data has been represented as n, %, Mean±SD.  A p-value≤0.05 is considered statistically significant.

Baseline demographics and clinical characteristics	Case Group (n=70)	Control Group (n=40)	p-value
Age (y)	65.73 ± 10.63	59.65 ± 10.29	0.039
Males, n (%)	50(71%)	24(60%)	0.013
Hypertension, n (%)	40(57%)	22(55%)	0.750
Diabetes Mellitus, n (%)	26(37%)	12(30%)	0.325
Smoking, n (%)	14(20%)	8(20%)	0.954
LVEF (%)	50.3056±7.8242	56.078±5.3987	0.031
Laboratory Data			
FSG (mmol/L)	7.38 ± 2.63	6.54 ± 1.82	0.521
Creatinine (mmol/L)	87.4 ± 75.14	86.81 ± 27.40	0.621
Haemoglobin (g/L)	132.2 ± 19.0	134.3 ± 17.3	0.653
WBC (×109/L)	9.24 ± 2.86	9.11 ± 2.89	0.798
TChol (mmol/L)	4.52 ± 1.32	4.95 ± 1.60	0.293
LDL-C (mmol/L)	2.69 ± 1.15	2.791± 1.29	0.842
HDL-C (mmol/L) 40 mg/dL (1.0 mmol/L)	42.28±6.62	56.82±6.98	0.043
TG (mmol/L)	1.71 ± 1.24	1.63 ± 0.99	0.056
CRP (mg/mL)	12.1 ± 7.1	11.1 ± 6.3	0.135
FGF21 (pg/mL)	166.599± 94.49791	54.13±48.467	0.034
Angiographic Severity Score in Case Group			
SYNTAX score	23.19±7.43		
Gensini score	50.03±27.30		

**Figure 1:**
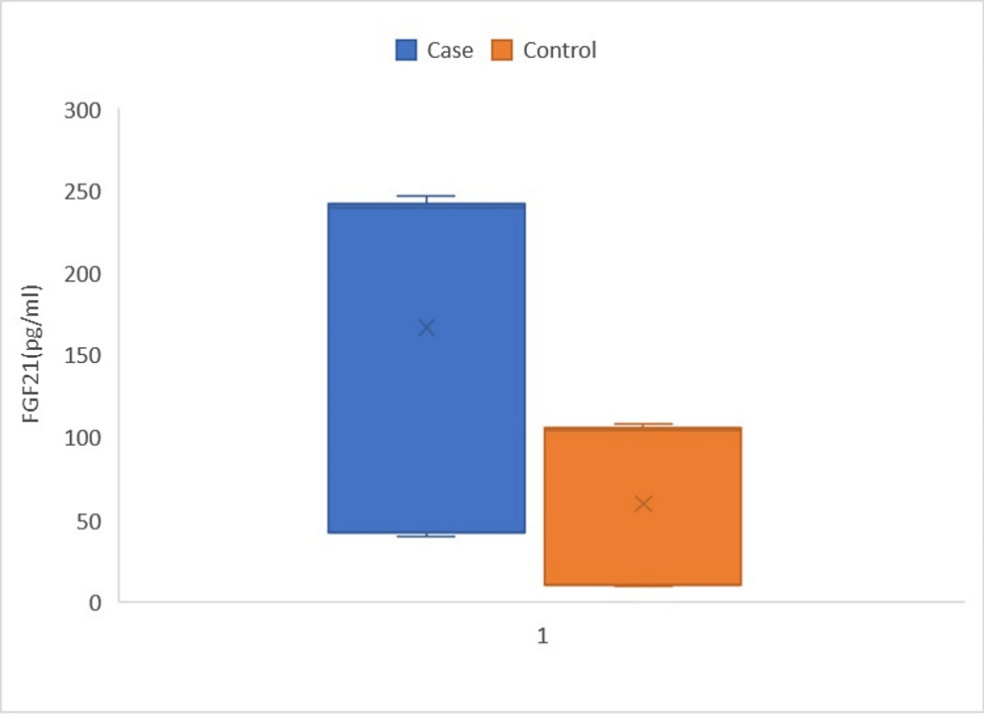
Box Whisker plot showing FGF21 levels in the case and control groups FGF21: fibroblast growth factor 21

The patients in the case group exhibited a substantially higher average age compared to those in the control group (65.73 ± 10.63 vs. 59.65 ± 10.29; p=0.039). In addition, the case group had a greater percentage of male patients compared to the control group (71% vs. 60%; p=0.013). The prevalence of cardiovascular risk factors, such as diabetes, hypertension, and smoking, was similar in both groups (P≥0.05). The LVEF exhibited a significant difference between the case and control groups, with mean values of 50.3056 ± 7.8242% and 56.078 ± 5.3987%, respectively (p=0.031). In addition, the group of cases exhibited lower levels of serum HDL-C (42.28 ± 6.62 vs. 56.82 ± 6.98; p=0.043). The levels of serum FGF21 were examined in both the case and control groups. The case group had considerably higher levels of FGF21 (166.59 ± 94.49791 pg/mL) compared to the control group (54.13 ± 48.467 pg/mL) (p=0.034), as shown in Figure 1.

**Figure 2:**
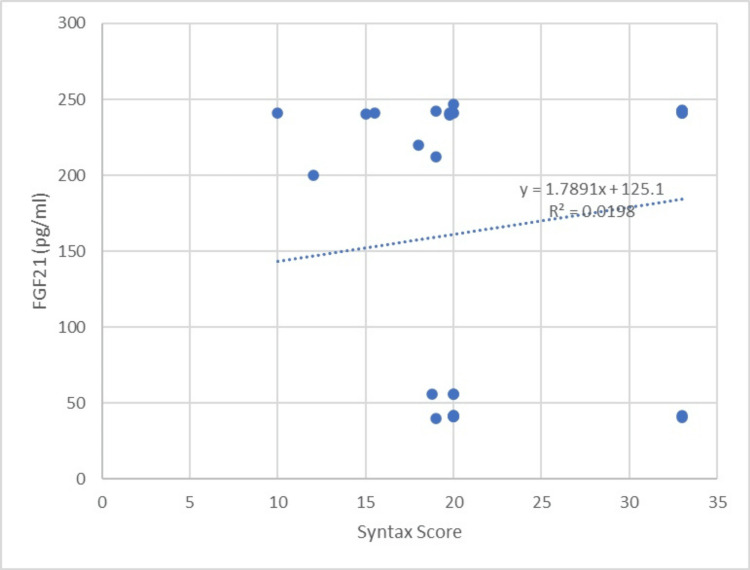
The graph showing the correlation between the FGF21 levels and the SYNTAX score FGF21: fibroblast growth factor 21

**Figure 3:**
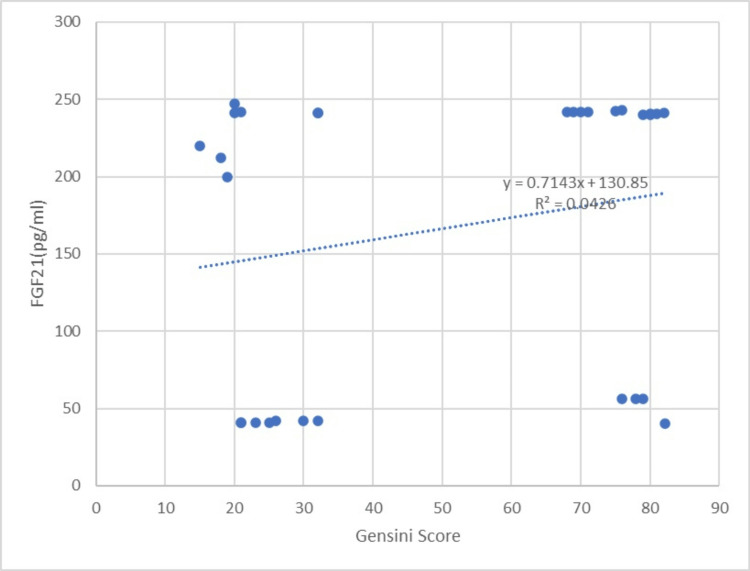
The graph showing the correlation between the FGF21 levels and the Gensini score FGF21: fibroblast growth factor 21

The levels of CRP were comparable in both groups, with values of 12.1 ± 7.1 mg/mL in one group and 11.1 ± 6.3 mg/mL in the other group (p=0.135). The case group had SYNTAX and Gensini scores of 23.19 ± 7.43 and 50.03 ± 27.30, respectively. Nevertheless, there was no statistically significant association between the risk assessments for CAD severity and the levels of FGF21 in the serum (correlation coefficient r=0.14070, p≥0.05, and r=0.206415, p≥0.05, respectively), as shown in Table 2, Figures 2,3.

**Table 2:** Showing correlation between FGF21 and SYNTAX and GENSINI score

FGF21	Correlation (r)
Syntax Score	0.1407
Gensini Score	0.2064

## Discussion

This study aimed to assess the role of FGF21 in the diagnosis and severity of CAD in this part of the world. Although we noticed significant variations in the blood FGF21 levels and the occurrence of CAD between the case and control groups, there was no evident correlation between the serum FGF21 levels and the severity scores.

Li et al [12] studied over 253 Chinese patients to find the association between serum FGF21 and mortality among patients with CAD. They concluded that high levels of FGF21 in the blood were an independent risk factor for CAD. Many other literature studies have demonstrated that levels of serum FGF21 may be elevated in individuals with CVD risk factors, including high body mass index (BMI), diabetes mellitus, and hypertension. However, the findings from the research on human beings to know the physiological roles of FGF21 have been inconclusive and inconsistent [13]. An et al [14] found in their study that levels of FGF21 were elevated in subjects with diabetes mellitus, and these elevated levels were related to the complications of diabetes mellitus, such as carotid plaques. In a different study with 235 patients, it was found that the group with CAD had elevated levels of serum FGF21 in comparison to the group without CAD [15]. We also observed that the levels of serum FGF21 were significantly different between the control and case groups, which is in accordance with other previous studies. Nevertheless, Lee et al [16] determined that serum FGF21 levels were not significantly different in persons diagnosed with coronary artery disease (CAD) using coronary CT angiography compared to those without CAD. Inflammatory and lipid indicators, low serum FGF21 levels, and age- and body-mass index-matched groups may account for this finding. This study's findings are in line with another study that looked at the role of FGF21 in SAP and found increased levels of FGF21 (SAP: 323.16 ± 434.66 vs. control: 266.46 ± 417.13 pg/mL; p=0.039). Nevertheless, multivariant regression analysis revealed that serum FGF21 levels cannot serve as a reliable indicator for SAP [17]. In another study, it was shown that patients with unstable angina pectoris had greater levels of serum FGF21 compared to the case and control groups. In contrast to our results, however, serum FGF21 levels were not significantly different between the case group and the control group [18]. This result may be due to variations in research design, variations in cardiovascular risk profiles among patients, diabetes mellitus, medication usage, BMI, distribution of visceral fat, and distribution of gender. Several studies have shown a correlation between the severity of CAD and serum FGF21.

Tucker et al [19] concluded in their review study, which summarized preclinical and clinical evidence, that FGF21 plays a function in HF. Preclinical studies have demonstrated that FGF21 has a role in the development of heart failure by mitigating oxidative stress, cardiac hypertrophy, and inflammation in cardiomyocytes. Nevertheless, the existing clinical evidence indicates that levels of FGF21 are unexpectedly elevated in HF, suggesting the presence of a state of FGF21 resistance similar to that observed in obesity. The determination of whether FGF21 has clinical significance as a biomarker is complicated by several potential confounding circumstances. The study conducted by Park et al [20] could not find any correlation between serum FGF21 levels and the severity of CAD as evaluated by SYNTAX scores. 

Kim et al [21] first observed a significant correlation between serum FGF21 levels and CAD severity scores in a cohort of 120 individuals. Nevertheless, after completing the final analysis, they determined that there was no correlation between FGF21 levels and the risk score in diabetes patients (r=0.332, p=0.055, and r=0.296, p=0.084, respectively). Consistent with prior studies, we also observed no association between serum FGF21 levels and CAD severity scores.

Indeed, Matuszek et al [22] validated that there is an inverse relationship between circulating FGF21 levels and HDL-C. They proposed that elevated FGF21 levels might be indicative of elevated insulin levels rather than elevated blood glucose levels. In their meta-analysis of studies, Lakhani et al [23] evaluated the roles of FGF21 levels in cardio-metabolic disorders. FGF21 can predict the incidence of CAD, the risks of obesity, diabetes mellitus, and renal disease development in diabetes mellitus patients. The novelty and strength of this study is that it was done for the first time in eastern Indian populations. The study was limited by its cross-sectional design, single-center nature, and relatively small sample size. The correlation analysis results may also be influenced by other confounding factors due to unmatched group characteristics like age, sex, personal habits like smoking, intake of alcohol, and other comorbidities.

## Conclusions

FGF21 is gaining recognition as a prospective addition to the FGF family, potentially playing a significant role in cardiovascular disease, particularly atherosclerosis. Statistically significant differences were seen in the serum FGF21 levels and the diagnosis of CAD between the case and control groups. Elevated levels of FGF21 are linked to the diagnosis of CAD in patients with SAP. However, there was no apparent correlation found between the serum FGF21 levels and the SYNTAX and Gensini scores. The role of FGF21 in the development of atherosclerosis and whether FGF21 could serve as a reliable marker needs to be studied further.

## Appendices

For details on the syntax score, please visit the web page at https://www.syntaxscore.org.
